# Comprehensive Analysis of Physicochemical Properties and Volatile Compounds in Different Strawberry Wines under Various Pre-Treatments

**DOI:** 10.3390/molecules29092045

**Published:** 2024-04-29

**Authors:** Zhenzhen Lv, Hui Liu, Wenbo Yang, Qiang Zhang, Dalei Chen, Zhonggao Jiao, Jiechao Liu

**Affiliations:** Zhengzhou Fruit Research Institute, Chinese Academy of Agricultural Sciences, Zhengzhou 450009, China; lvzhenzhen@caas.cn (Z.L.); liuhui@caas.cn (H.L.); yangwenbo@caas.cn (W.Y.); zhangqiang02@caas.cn (Q.Z.); chendalei@caas.cn (D.C.); jiaozhonggao@caas.cn (Z.J.)

**Keywords:** pre-fermentation, maceration, thermovinification, enzymatic hydrolysis, physicochemical properties, volatile aroma compositions

## Abstract

Pre-fermentation treatment has an important impact on the color, aroma, taste, and other characteristics of fruit wine. To discover suitable pre-treatment techniques and conditions that yield strawberry wine of excellent quality, the influences of juice fermentation, pulp maceration, thermovinification, and enzymatic hydrolysis pre-treatments on the basic chemical composition, color, antioxidant capacity, and volatile organic compounds in strawberry wines were investigated. The results showed that the color, antioxidant properties, and volatile aroma of strawberry wines fermented with juice were different from those with pulp. Strawberry wines fermented from juice after 50 °C maceration had more desirable qualities, such as less methanol content (72.43 ± 2.14 mg/L) compared with pulp-fermented wines (88.16 ± 7.52 mg/L) and enzymatic maceration wines (136.72 ± 11.5 mg/L); higher total phenolic content (21.78%) and total flavonoid content (13.02%); enhanced DPPH (17.36%) and ABTS (27.55%) free radical scavenging activities; richer essential terpenoids and fatty acid ethyl esters, such as linalool (11.28%), ethyl hexanoate (14.41%), ethyl octanoate (17.12%), ethyl decanoate (32.49%), and ethyl 9-decenoate (60.64%); pleasant floral and fruity notes compared with juice-fermented wines macerated at normal temperatures; and a lighter color. Overall, juice thermovinification at 50 °C is a potential pre-treatment technique to enhance the nutrition and aroma of strawberry wine.

## 1. Introduction

Strawberry is a rose family of herbal berries with red fruit. This fruit and its derived products are considered rich in polyphenols, flavonoids, anthocyanins, and other antioxidant-active substances [1,2], and offer multiple health benefits, including antioxidant, cardiovascular, antihypertensive, and antiproliferative effects [3]. Although strawberry is widely consumed as a fresh product, currently, there are more and more processed products commercially available, such as jams, freeze-dried strawberries, alcoholic and nonalcoholic beverages, etc. [4,5,6]. Strawberry wine is a kind of deeply processed product, fermented by yeast and brewed. The transformation of a highly perishable fruit into a beverage increases its conservation period and prevents food waste.

In order to improve the quality of wine and give it an elegant aroma and pleasant taste, many scholars have conducted research on the influence of pre-fermentation treatment technology on wine quality. Pre-fermentation treatment refers to the different treatments of fruit raw materials before fermentation [7], so that the aroma, anthocyanins, and nutrients in raw materials dissolve differently, and produce different changes in the fermentation process, which has an important impact on the color, aroma, taste, and other characters of fruit wine. Traditional white vinification utilizes clear juice after removing the skin and seeds [8]. In contrast, traditional red vinification utilizes skin maceration without removing the solids from the juice, and the skin contact can increase the polyphenol concentrations apparently by continuous extraction during fermentation [9]. At the same time, other pre-fermentation techniques, such as thermovinification and pectin enzymatic hydrolysis, are becoming more popular in the wine industry. The methanol content and aroma component in fruit wine can be affected by juice and pulp fermentation, as well as enzyme treatment [7,10]. During thermovinification, destemmed grapes are heated up to temperatures ranging from 60 or 87 °C in order to disrupt the cell structure and initiate an instant extraction of all cell constituents. The holding time of the heated pulp varied between two minutes at 87 °C to several hours at lower temperatures [11]. Compared with wine produced by traditional processes, the red wine produced by thermovinification has a bright color, rich aroma, and harmonious taste, and is more mature [12,13]. Pectinases, hemicellulases, or cellulases may be also used in vinification for enzymatic hydrolysis of cell walls [14] and for the degradation of pectin substances. As described by Paranjpe et al. [15], the enzymes may destabilize anthocyanins by affecting their deglycosylation. However, pre-fermentation treatment techniques and their conditions must be chosen individually for each kind of fruit wine to achieve optimal characteristics, because the yield of the target compounds depends on an optimal combination of these.

To sum up, numerous studies have been conducted to investigate the phenolic profile, antioxidant capacity, and volatile compounds in strawberries [2,6]. In brewing technology, Sun et al. [16] explored the effects of sulfur dioxide and maturation on the aroma, physical–chemical, and sensory quality of strawberry wine. There are few reports about the influences of different pretreatment methods, such as juice fermentation, pulp maceration, thermovinification, and enzymatic hydrolysis pretreatment, on the chemical composition, nutrition, color, and aroma of strawberry wine. For this reason, the objective of the study was to investigate the impacts of different pre-treatment approaches on the basic chemical composition (content of ethanol, methanol, total sugar, sugar-free extract, titratable acidity, and organic acid), color, antioxidant capacity, and volatile organic compounds to discover suitable pre-fermentation treatment techniques and conditions that yield strawberry wine of excellent quality.

## 2. Results and Discussion

### 2.1. Chemical Compositions and Color Properties

Six pre-fermentative treatments were applied before alcoholic fermentation, as shown in Table 1. The chemical indicators and the color attributes of strawberry wines fermented by different pre-treatment methods were presented in Table 2. These strawberry wines exhibited similar levels of alcohol content (12.17–12.50%) as a result of the initial sugar content adjustment to the same level (24%) before fermentation. However, the alcohol content of PJ-fermented strawberry wine was the highest (*p* < 0.05), which may be because the sugar and other nutrients in strawberry were extracted more effectively, thus being more accessible after pectinase treatment. Except for PJ strawberry wine, other wines all belonged to the dry type, based on total sugar contents below 4 g/L. The contents of sugar-free extract in strawberry wines fermented and kept on pulp (NM, PM) were higher than those of wines fermented from juice, suggesting an enhancement of extraction of soluble substances from strawberry pulp. Compared to no pretreatment (CJ), maceration was favored to increase the content of the sugar-free extract. More soluble solids, organic acids, polysaccharides, and phenolic substances in strawberries could be soaked out through maceration.

Typically, commercial pectinase preparation includes pectinesterase, polygalacturonase, and pectinlyase. The hydrolysis of methyl ester groups in pectin by pectinesterase led to a notable increase (*p* < 0.05) of methanol content in the PJ and PM final wines by 1.52–15.41 times compared to those without pectinase, which was confirmed by the research of Wei et al. [17]. However, these amounts (136.72 mg/L and 134.08 mg/L) did not exceed the limit set by the International Office of Vine and Wine (OIV) at <400 mg/L for red wines and <250 mg/L for white or rose wine (International Organisation of Vine and Wine, 2015). The CJ wine sample made from juice without maceration showed the lowest methanol content, and the NJ, 50J, and NM samples followed in turn. These sources of methanol were mainly due to the activation of endogenous pectinesterase, which occurs naturally in fruits and is responsible for the release of methanol in fruit juices and wines without the addition of pectinase [18].

The organic acid profile is highly associated with the acidity of fruit wines and is an influential factor in its flavor, which can be influenced by the fruit, yeast strain, and fermentation conditions. Five organic acids were determined in strawberry wines, including citric, malic, succinic, lactic, and acetic acid. As shown in Table 2, citric acid was the primary organic acid in strawberry wines, accounting for 59.72–71.52% of the total organic acids. Some reports have indicated that citric acid is the predominant organic acid in strawberry fruit [19,20]. Malic acid was the second most abundant organic acid, followed by succinic acid, lactic acid, and acetic acid, in all samples. Liu et al. [21] reported a significant increase in malic acid and succinic acid in persimmon wine with pectinase pretreatment, which was not observed in the present result. The concentrations of lactic and acetic acid in pulp-fermented strawberry wines (NM, PM) increased by 34.21–63.83% and 41.24–91.67%, respectively, compared with juice-fermented strawberry wines (CJ, NJ, 50J, PJ). However, acetic acid is the main component of volatile acid, which is a “barometer” in the wine storage process. Reports on pungent wine have shown that high levels of acetic acid lead to increased bitterness and poorer flavor. The acetic acid content in fruit wines is normally 0.1–0.5 g/L, and when it exceeds 0.8 g/L, rancidity will appear [22]. Based on the acetic acid content, it could be inferred that the fermentation method with pulp (NM) was not appropriate for making strawberry wine. Juice-fermented strawberry wines (CJ, NJ, 50J) tended to contain decreased acetic acid (0.10–0.12 g/L) compared with wild strawberry wines fermented with mashes (0.71 g/L) [23]. During the fermentation with pulp, fruit residue accumulates on the surface of the wine and forms a wine cap, which is not conducive to heat dissipation, resulting in a higher temperature and increased content of lactic acid and acetic acid. 

The CIELAB parameter lightness (L*) is negatively correlated with color intensity. The positive values of a* and b* are related to the degrees of red and yellow, respectively. Generally speaking, the wine’s color and appearance quality both improve with increasing values of a* and b*. The CIELAB parameter chroma (c*) and hue angle (h) are the corresponding angular coordinates derived from the Cartesian coordinates a* and b*, but they are better related to the human sensory perception of color. The higher the c* value is, the deeper the color saturation. The closer h is to 0, the redder the color is, and the closer it is to 90, the more yellow the color is. CJ and PJ strawberry wines had a lower L*, but higher a*, b*, and c*, which indicated that the two types of wines were bright in color intensity and high in saturation. The h of the CJ wine sample was 43.56, distributed in the orange-red region. The h values of PJ and NJ samples were 53.63 and 54.64, respectively, in the orange-yellow region. The h values of NM, PM, and 50J samples ranged from 59.85 to 61.80 in the yellow region, and were significantly higher than the other three wines (*p* < 0.05); meanwhile, a* values (15.60–16.35) were lower (*p* < 0.05). The results indicated that fermentation with pulp and high-temperature maceration might lead to the color of strawberry wine being more yellow and lighter; in other words, these two treatments were not conducive to preserving the red color in strawberry wine. This color appearance may be related to lower contents of anthocyanin and derived pigments. Anthocyanins are susceptible to and easily affected by temperature and oxygen [24]. Conventional pulping and thermal treatment usually have negative impacts on the color and anthocyanins of fruit pulp [25,26].

### 2.2. Total Phenolic Compounds and Antioxidant Capacity

**Figure 1:**
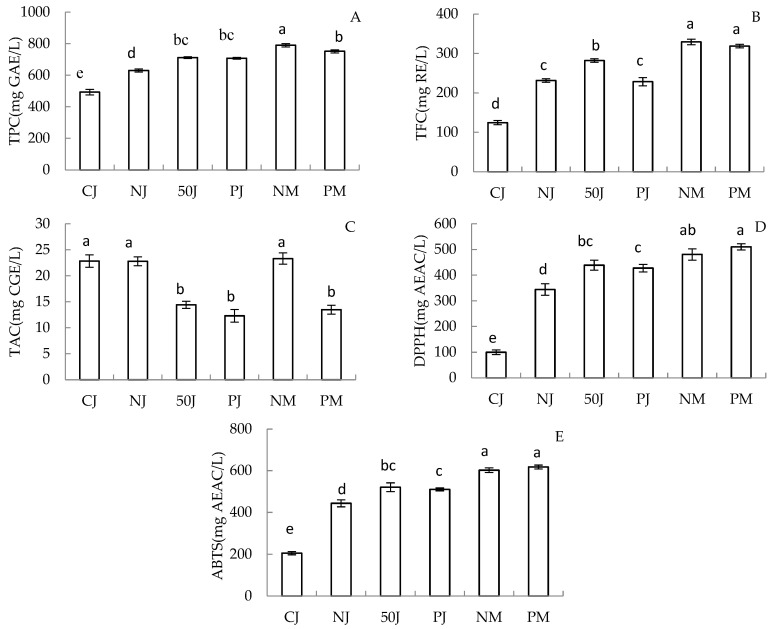
Total phenolic (**A**), total flavonoid (**B**), total anthocyanin (**C**), and scavenging ability of ABTS (**D**) and DPPH (**E**) free radicals of 6 strawberry wines fermented by different methods. Values identified by the same letters were not significantly different at the 0.05 level (one-way ANOVA–Dunace).

The total phenolic (TPC), flavonoid (TFC), and anthocyanin (TAC) contents, as well as the scavenging ability of ABTS and DPPH free radicals, of six fermented strawberry wines are shown in Figure 1A–E. The TAC in CJ strawberry wine was significantly higher, but the TPC and TFC were the lowest. A similarly low result was observed for the scavenging ability of ABTS and DPPH free radicals in CJ strawberry wines. This suggested that fermentation from juice without maceration was favorable for the extraction and preservation of anthocyanin in strawberry wine production, but not for the leaching of other phenolic substances. The TPC, TFC, and scavenging ability of ABTS and DPPH free radicals of the NJ and 50J strawberry wines were 12.33–25.42%, 23.45–42.18%, 20.77–35.81%, and 22.08–39.68% lower, respectively, than those values of NM wines fermented with pulp, which is significant. It was indicated that the vinification of juice could not replace the traditional fruit pulp steeping fermentation, as more phenolic substance could be transferred from pulp to strawberry wine. Compared to NJ, thermovinification at 50 °C (50J) increased the TPC by 21.78% and the TFC by 13.02%, respectively. Similarly, increases were discovered in the scavenging ability of ABTS and DPPH free radicals, where the increase rates were 27.25% and 11.28%, respectively. Inconsistently, thermovinification noticeably decreased the TAC of the 50J sample from 22.79 ± 0.87 mg CGE/L to 14.41 ± 0.68 mg CGE/L compared with maceration at a normal temperature (NJ). These findings are in agreement with the red wine study, which resulted in similar TPC, TFC, and TAC of wine samples fermented through thermovinification [27,28].

The TPC, TAC, and scavenging ability of ABTS and DPPH free radicals in 50J strawberry wine were higher than those in PJ ones, but there was no significant difference between them. The TFC of PJ strawberry wine added with pectinase was less than that of 50J wine. However, there are wines produced via enzymatic pre-treatment which have shown higher concentrations of total polyphenols and anthocyanins [27].

### 2.3. Volatile Organic Compounds (VOCs)

**Figure 2:**
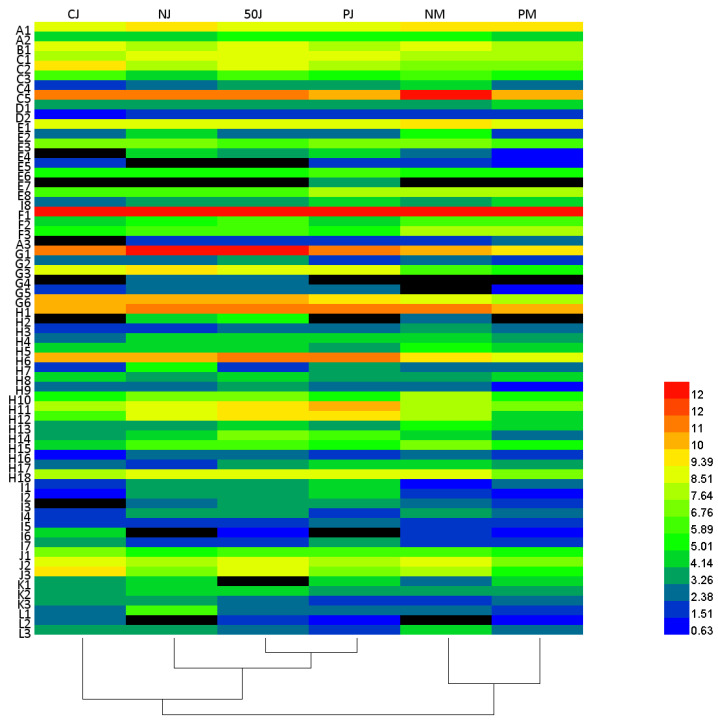
Heatmap of VOCs in strawberry wines. The red blocks indicate the up-regulated compositions, and blue blocks indicate the down-regulated compositions.

**Figure 3:**
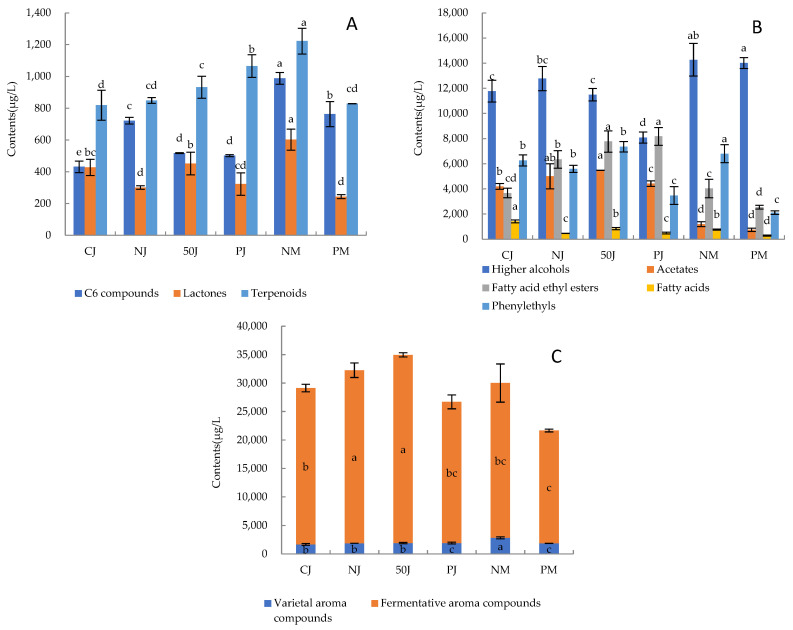
The total relative concentration of volatile compounds within the different aromatic families: varietal aroma compounds (**A**), fermentative aroma compounds (**B**), and total aroma compounds (**C**). Values identified by the same letters were not significantly different at the 0.05 level (one-way ANOVA–Dunace).

Despite strawberry volatile compounds having been studied extensively [29], the information published to date on the volatiles of strawberry wine is still in short supply. In this study, a total of 63 VOCs were identified and quantified in strawberry wines by using headspace HS-SPME-GC-MS, which was divided into two kinds: the varietal aroma included two C6 compounds (A1–A2), one lactone (B1), two furans (C1–C2), eight terpenoids (D1–D8), and one phenolic acid ester (E1), while the fermentative aroma included four higher alcohols (F1–F4), five acetates (G1–G5), fourteen fatty acid ethyl esters (H1–H14), six other esters (I1–I6), three fatty acids (J1–J3), three aldehydes and ketones (K1–K3), eleven phenylethyls (L1–L11), and three others (M1–M3) (Table S1). The differences in the VOCs in strawberry wines fermented using various methods are indicated using a heatmap (Figure 2), and the relative concentration histograms of various kinds of VOCs in different samples are presented in Figure 3A–C. The total concentrations of detected volatile aroma components for the six strawberry wines fell between 22.49 and 35.17 mg/L (Figure 3C), mainly including phenyl ethyl (5.18–16.28%), isobutanol (1-Butanol, 3-methyl-) (29.96–63.45%), isobutanol acetate (3.23–14.06%), 2-phenyl ethyl acetate (1.09–6.44%), ethyl caproate (hexanoic acid ethyl ester, 5.40–12.94%), and ethyl caprylate (octanoic acid ethyl ester, 2.17–9.99%), as shown near the red blocks (Figure 2, Table S1). However, compounds with high content may not necessarily make a significant contribution to wines, which cannot be detected by people with high olfactory thresholds, so the odor activity values (OAVs) of aroma compounds were calculated in this research. Aroma compounds with OAVs greater than 1 directly contribute to the aroma of wine, and the coordinating contribution of compounds with low OAVs between 0.1 and 1 to aroma quality has been confirmed by researchers [30]. Table 3 shows 33 probable odor-active ingredients with OAVs greater than 0.1. The higher the values, the more significant the contribution of the t substance to the aroma.

#### 2.3.1. Varietal Aroma Compounds

In general, terpenoids with unique aromas and flavors are the main variety of aroma compounds and can add complex flavors and layers to a fruit wine [45]. Eight kinds of terpenoids were identified in six strawberry wines, accounting for 42.96–54.92% of the varietal aroma compounds and 2.63–4.07% of the total aroma compounds (Table S1). The relative contents of linalool, α-terpinol, and trans-nerolidol (1, 6, 10-Dodecatrien-3-ol, 3, 7, 11-trimethyl-, (E)-) were relatively high, accounting for 53.96–74.33%, 8.01–14.43%, and 7.52–26.26% of terpenoids, respectively. Linalool was the primary contributor of fruity and floral aromas to strawberry wines, the OAVs of which were 34.45–52.78 (Table 3). The OAVs of myrtenol in NJ, 50J, 50PJ, and NM strawberry wines were 1.13–2.95 (Table 3). It has a floral and minty aroma and has been reported as the characteristic aroma of blueberry [36] and blackberry [46]; hence, it was also the characteristic aroma in those wines. Terpenes are affected by many concurrent processes taking place during mash fermentation: their extraction from fruit skins, hydrolysis of bound forms, conversions induced by yeasts, and loss by adsorption onto solids [47]. NM strawberry wine contained the highest level of terpene compounds (1222.53 µg/mL) (Figure 3A) and total terpene OAVs (Table 3), followed by PJ (1064.75 µg/mL) and 50J (931.98 µg/mL) strawberry wine (Figure 3A); however, the total OAVs of terpenoids in the 50J strawberry wine were higher than that of PJ (Table 3). The other three samples had levels below 800 µg/mL. As a result, it might be that the three treatments of fermentation with pulp, thermal maceration, and enzymatic hydrolysis promote the release of terpene precursors from the skins, and then further release these compounds via acidolysis or enzymatic hydrolysis during the fermentation process. However, the contents of terpenes in fermented strawberry wine with skin were not all high, and the contents of PM strawberry wine fermented with skin and pectinase were reduced to 828.44 µg/mL. The reasons for these results are worth exploring in the future.

The second highest relative content variety aroma component was C6 compounds, accounting for 25.43–40.60% of varietal aroma compounds and 1.48–3.52% of total VOCs (Table S1). The OAVs of two out of three C6 compounds (hexanol and nonanol) were around 0.1, which were the main contributors to the green aroma in berries and wines, and could result from the enzymatic degradation of unsaturated fatty acids in the berries and glycosidic aroma precursors [45]. Their concentration depends on the variety, ripeness stage, treatments prior to fermentation, and temperature/duration of contact with the skins. Similarly to those described for terpenoids, must maceration increased the relative content of this type of compound by 29.43–128.96% in NM strawberry wines compared to other samples (Figure 3A). A significantly higher relative concentration of C6 compounds was found in NM and PM wines (Figure 3A), as a consequence of more oxygen being dissolved in the aqueous medium during the pre-fermentation step, which favored the formation of C6-aldehyde precursors by the LOX/HPL pathway [48]. Red wines produced by thermovinification (pre-fermentation, 20 min at 75 °C) exhibited lower amounts of C6-compounds in relation to those fermented at normal temperature as a result of the inactivation of lipoxygenase activity by heat [15], and the same was found in this experiment in 50J wines (Figure 3A).

Only one type of lactone compound was detected, γ-decanolide, accounting for 12.96–25.20% of varietal aroma compounds and 0.93–2.00% of total VOCs (Table S1). As its OAV was 24.35–60.19 (Table 3), it was one of the key aroma compounds of strawberry wine. According to reports, multiple strawberry varieties contain lactone compounds, which are the specific source of the peach aroma in strawberries [49]. Similarly, must maceration increased the relative content of lactone 33.27–147.16% in NM strawberry wines, compared to other samples (Figure 3A). This suggested that prolonged maceration and fermentation with the skin promoted the extraction of lactone from strawberry pulp into strawberry wine.

Among a variety of other aromas, methyl salicylate was the main odor-active compound, as its OAV was 0.16–0.80 (Table 3). Methyl salicylate has been reported as a characteristic aroma of blueberry fruits [50], which may provide fresh fruit flavor to fruit wines.

#### 2.3.2. Fermentative Aroma Compounds

In the fermentation aroma, higher alcohols (mainly isobutanol) occupied the highest relative content level, reaching 8.08–14.27 mg/L (Figure 3B). A more relative content of fusel and other alcohols of about 11.72–76.56% was exhibited by NM and PM samples produced with pulp compared to that of strawberry wines fermented from juice (CJ, NJ, 50J, PJ) (Figure 3B). Other authors have also reported higher concentrations of these compounds in red wines obtained by enzyme maceration [47] or by pre-fermentative maceration at 20 °C [51]. The authors correlated the obtained results mainly with an intense extraction of amino acids, which are the main precursors for fusel alcohols [47]. The fermentation with pulp treatments favored the release of different volatile precursors, among them amino acids. It is known that fusel alcohol concentrations of less than 400 mg/L provide a desired floral complexity to the wine [47]. Therefore, the higher levels of these compounds in NM and PM strawberry wines fermented with pulp was considered to be positive. Although higher alcohols were present in higher concentrations, their olfactory thresholds were usually higher, so they might have had less of an effect on the overall aroma than other aromas in fermented strawberry wines.

As a very important component of wine aroma, esters can make a positive contribution to the quality of wine, provide delicate “fruity” and “floral” odors, and affect the sensory properties and aromatic finesse of the wine [48,52]. Their types and contents were relatively abundant in the strawberry wine, accounting for 15.29–47.44% of the total aroma compounds, among them acetic esters 3.43–16.55%, ethyl esters 11.75–30.63% and other esters 0.10–0.27% (Table S1). The relative contents of isoamyl acetate, hexyl acetate, ethyl caproate, ethyl caprylate, and ethyl caprate were higher than their olfactory thresholds, and their OAVs were greater than 1, especially ethyl caproate and ethyl caprylate, which could reach 112.47–1333.33 (Table 3). This contributed apple, pineapple, and strawberry flavors to strawberry wine and were the main characteristic aroma substances.

The highest relative concentrations of major acetates and ethyl esters were found in the 50J sample (Figure 3B, Table S1). Ethyl hexanoate, ethyl octanoate, ethyl decanoate, and ethyl 9-decenoate increased by 14.41%, 17.12%, 32.49%, and 60.64% compared to NJ, followed by PJ strawberry wine, which is in accordance with other findings regarding wines obtained through thermal treatment and liquid fermentation [10,11,27]. Geffroy et al. [27] explained this as a consequence of the absence of solids and the high rate of yeast-assimilable nitrogen extraction during pre-fermentation thermal treatment. Fermentation with pulp did not favor the formation of acetates or ethyl esters, since these wines always showed lower total concentrations of these compounds than wines fermented from juice (CJ, NJ, 50J, PJ) (Figure 3B). Fischer et al. [11] attributed it to the lack of mechanical treatments, which diminished ester evaporation. Callejón et al. [53] observed an inhibiting effect of skins on the formation of linear esters and acids. Lukic et al. [10] assumed that the skins either provided competitive substrates or enzyme inhibitors or adsorbed these compounds.

However, the results found in the literature about the effect of these techniques on ethyl ester content are not always concordant, not allowing us to determine the general effect of maceration techniques on the contents of these compounds. For example, Hernandez et al. [54] found lower levels of ethyl butanoate, ethyl hexanoate, and ethyl decanoate, but higher levels of diethyl succinate, in white wines with enzymatic maceration. Similarly, Alvarez et al. [55] obtained cold-macerated wines with lower diethyl succinate but higher ethyl butanoate than the control wines. On the other hand, Albanese et al. [56] reported that ethyl decanoate levels increased when Shiraz wines were produced using prior cryo-maceration.

Fatty acids are the main volatile compounds in fruit wine that contribute to the complexity of aroma and balance of taste, and have been described as fruity, cheesy, fatty, and rancid notes. However, high concentrations of acids can lead to negative flavors such as fat and spoilage. Three fatty acid compounds were identified in strawberry wines: hexanoic acid, 2-methyl-hexanoic acid, and octanoic acid. Among them, the contents of hexanoic acid and octanoic acid were high (Table S1), and their OAVs ranged from 0.09 to 1.65 (Table 3). The different treatments utilized gave different results. CJ strawberry wine fermented from juice without any treatment had the highest levels of fatty acids. 50J and NM strawberry wines followed (Figure 3B). It was found that the composition and fermentation conditions of grape juice have a significant impact on the content of fatty acids [57].

Phenylethyl compounds in wine samples come from both varietal aroma and fermentation aroma. By comparing the contents of various components of phenylethyl compounds in wine samples and grapes in the literature, it was found that the contents of these compounds in grapes are much lower than in wine [58,59], which means that most of them are produced by alcohol fermentation. Therefore, phenylethyl compounds were classified as fermentation aromas in this study. Phenylethyls were the third class of substances with high relative content in strawberry wines, accounting for 9.38–22.68% of total VOCs (Figure 3B, Table S1), besides high alcohols and esters. The contents of phenylethanol, phenylethyl acetate, benzaldehyde, ethyl cinnamate (2-Propenoic acid, 3-phenyl-, ethyl ester), and styrene were relatively high, but the contents of phenylethanol and benzaldehyde were lower than the olfactory threshold, and the contents of phenylethyl acetate, ethyl cinnamate, and styrene were higher than the olfactory threshold (Figure 2, Table 3 and Table S1). Thus, these three compounds contributed significant rose, floral, and honey aromas to strawberry wines. The OAV of ethyl cinnamate reached 155.73–516.99, which was reported to be the characteristic ester of strawberry varieties and their wines [60]. The highest relative concentration and OAVs of major phenylethyls were found in 50J strawberry wine (Table 3). Strawberry wines fermentated with pectinase were not conducive to the formation of phenylethyls, because PJ and PM strawberry wine reduced the contents of these compounds compared with 50J and NM strawberry wines, except ethyl phenylpropionate and ethyl cinnamate (Figure 3B). Other authors have also described lower concentrations of phenylethyls in red wines obtained by adding pectolytic enzymes [52]. Saccharomyces cerevisiae decomposes aromatic amino acids such as phenylalanine and tyrosine to produce phenylethanol and phenylethyl acetate. It may be that the enzyme operation provides a competitive substrate for cerevisiae to metabolize aromatic amino acids, thus reducing the metabolism of this class of amino acids, resulting in a significant reduction in phenylethanol and phenylethyl acetate.

In addition, volatile aroma substances of furans, aldehydes, ketones, other esters, and aromatic species were also detected in the six wine samples, and the contents of these volatile substances were low. They played a complementary and modifying role in the aroma of the strawberry wines.

#### 2.3.3. Principal Component Analysis (PCA)

**Figure 4:**
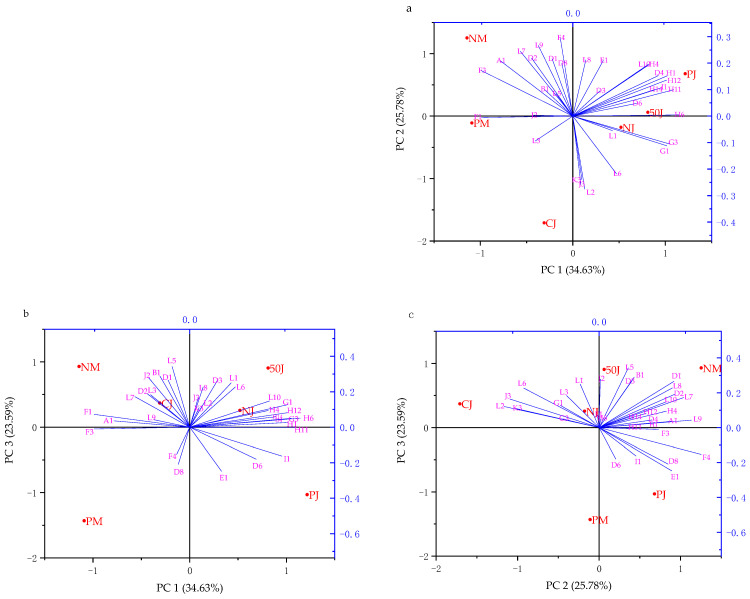
Principle component analysis (PCA) of volatile compounds in strawberry wines. (**a**) PCA-biplot of the store and loading PC1-2. (**b**) PCA-biplot of the store and loading PC1-3. (**c**) PCA-biplot of the store and loading PC2-3.

To compare the relative differences in VOCs of strawberry wines obtained using different fermentation methods, a PCA analysis was performed according to OAVs above 0.1 of 34 selected VOCs, as shown in Table 3 (Figure 4). Five principal components were established, and PC1, 2, and 3 contributed 83.99%, which could better reflect the variance of the original data. As the PCA-biplot of the store and loading showed in PC1, 2, and 3 (Figure 4a–c), samples in different methods of fermentation could be separated obviously. The NM and PM strawberry wines fermented with pulp had higher scores in the PC1 negative direction; meanwhile, NJ, 50J, and PJ strawberry wines produced from juice were grouped centrally in the positive half-axis of PC1 (Figure 4a,b). NM and PJ strawberry wines were loaded in the positive vector of PC2. CJ strawberry wines tended to score higher in the negative direction in PC2 (Figure 4a,c). PJ and PM strawberry wines fermented with enzymes were distinguished from other strawberry wines due to having higher scores in the negative half-axis of PC3 (Figure 4b,c).

Compounds negetively loaded on PC1 were higher alcohols (1-octanol, isoamyl alcohol, and 2-nonanol), C6 compounds (1-hexanol), acids (hexanoic acid), phenylethyls (ethyl benzoate, ethyl phenylpropionate, acetophenone, and phenylethyl alcohol), γ-decanolactone, and several terpenoids (geranyl acetone, linalool, and trans-nerol), mainly contributing spicy, whiskey, green, and weak flower aromas. Ethyl esters (ethyl caprylate, ethyl decanoate, ethyl 9-decenoate, ethyl caproate, ethyl laurate, and ethyl enanthate), acetates (hexyl acetate and isoamyl acetate), terpenes (myrtenol and farnesol), and phenylethyls (ethyl phenylacetate and ethyl phenylpropionate) were positively loaded on PC1, mainly contributing fruity and flowery aromas. Terpenoids (linalool, geranyl acetone, trans-nerol, myrtenol, and α-terpinol), phenylethyls (ethyl benzoate, ethyl phenylacetate, ethyl phenylpropionate, and ethyl cinnamate), γ-decanolactone, 1-octanol, and 2-nonanol were mainly loaded in positive PC2, contributing plenty of flowery, sweet aromas and some fruity aromas. The key substances to the negative PC2 direction were benzaldehyde, capric aldehyde, caprylic acid, and phenyl ethyl acetate, which primarily contributed to bitter almond, cheese, and fatty aromas. Phenylethyls (phenylethyl acetate, phenylethanol, ethyl benzoate, benzaldehyde, acetophenone, styrene, and ethyl phenylacetate), acids (octanoic acid, hexanoic acid), γ-decanolactone, α-terpinol, and linalool had positive effects on PC3, contributing rose, fat, sour, cheese, and flower flavors. Methyl salicylate, trans-nerolidol, 2-nonanol, methyl caprylate, and farnesol, with peppermint, orange, citrus, and lemon aromas, were negative vectors of PC3.

In general, the NM and PM wine samples shared more prominent whiskey characteristics, while the NM wine tended to have a floral, fruity, and sweet aroma, and the PM wine tended to have citrus and orange flavors and less northern fruity aromas, such as apple and pear. The overall flavor of CJ strawberry wine was relatively weaker compared to other strawberry wines fermented from juice, focusing on rose, almond, and cheese characteristics. The 50J and PJ strawberry wines had more sweet fruit, flower, and honey aromas than the NJ wine sample, whereas the difference between them was that the 50J wine showed more rose and cheese aromas, and the PJ wine had more orange and lemon characteristics.

## 3. Materials and Methods

### 3.1. Raw Material and Reagents

The experiment was performed with the native and the most widespread strawberry variety (Sweet Charlie) from Zhengzhou during the harvest of 2019. The soluble solid content, total acidity, and pH of ripe strawberries at harvest were 10.8%, 10.7 g/L (as tartaric acid), and 3.03. In this work, the strawberries were harvested and collected in plastic cases of 2.5 kg capacity each.

Organic acid standards with purity > 99% were purchased from Dr. Ehrenstorfer GmbH Co. (Augsburg, Bavaria, Germany). Other chemical standards, including gallic acid; rutin; cyanidin-3-O-glucoside; ascorbic and 2-octanol; Folin–Ciocalteu reagent; 1, 1-diphenyl-2-picrylhydrazyl (DPPH); and 2, 2′-azinobis-(3-ethylbenzothiazoline-6-sulfonic acid) (ABTS) with purity > 98% were purchased from Sigma-Aldrich Co. (St. Louis, MO, USA). (NH_4_)_2_HPO_4_ analytic chemicals were purchased from Shanghai Macklin Biochemical Co. (Shanghai, China). Water was purified using Milli-Q Academic (Millipore, Molsheim, France).

### 3.2. Winemaking and Maceration Techniques

All of the vinifications were performed in 5 L glass jars with 5 randomly selected cases of strawberries. After berry pedicles were removed, strawberries were crushed, mashed, and sulphurated (0.1 g/L of potassium metabisulfite). All treatments were performed in duplicate.

**Figure 5:**
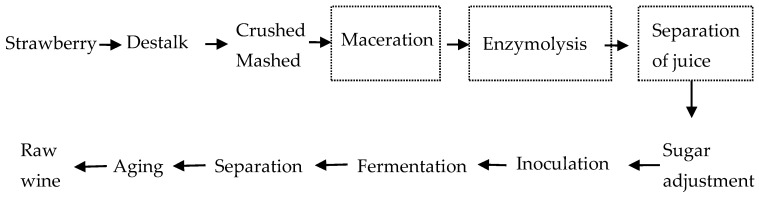
Winemaking procedure scheme. Notes: The procedure in the dotted box depended on the specific pre-treatment process in Table 1.

Destemming and must crushing was carried out via the addition of 0.1 g/kg of a commercial pectinase (>40 PA/mg, Darmstadt, Germany), and this was performed at 50 ± 1 °C for 3 h. After maceration, the juice was pressed from the must with two layers of gauze and transferred into another tank. Mashes or juices were added with 160 g/kg of sugar, inoculated with wine yeast seed at 5% *v*/*v*, and fermented at 22–24 °C for 10 days. After fermentation, the wine was separated by filtration with two layers of gauze and then stored at 15–20 °C for another 3 months before further analysis. For the preparation of the EC-118 (Lallemand SA, Montreal, QC, Canada) yeast seed, it was stored on a laboratory-inclined surface and inoculated in sucrose solution (5% *w*/*v*) at 28 °C for 24 h, then added into strawberry juice at 28 °C for 24 h. Cell count at inoculation was 2 × 10^8^ CFU/mL. A diagram of the process is shown in Figure 5.

### 3.3. Determination of Physicochemical Indicators and Color

The fermentation broth was centrifuged at 4 °C and 10,000 r/min for 10 min, and the supernatant was taken for determination. The determination of alcoholic, sugar-free extract, total sugar, titratable acidity, and methanol content referred to the People’s Republic of China of GB/T 15038-2006: “General Methods of Analysis for Wine and Fruit Wine” [61]. The color of the wine, including L* (brightness), a* (greenness [−] to redness [+]), b* (blueness [−] to yellowness [+]), c* (color saturation), and h (hue), was determined using a Chroma-Meter (Minolta CM5, Osaka, Japan). The samples were measured against a white ceramic reference plate. All measurements were run in triplicate.

### 3.4. High-Performance Liquid Chromatography (HPLC) Analysis of Organic Acids

Organic acids were analyzed by previously used methods [62] with some modifications. The pretreatment of the sample was the same as Section 3.3, and the supernate was filtered with a 0.22 μm microfilter. Organic acids were analyzed using an e2695 HPLC system equipped with a UV-2489 detector (Waters, Wilford, MA, USA). Chromatographic peaks were separated by a Waters X Select^®^ HSS T3 column (4.6 × 250 mm, 5 μm). (NH_4_)_2_HPO_4_ solution (0.02 M, pH 2.4) was performed at a rate of 1 mL/min at 30 °C, and chromatograms were detected at 210 nm. Organic acids were identified and quantified by comparing the relative retention times and peak areas of the samples and standard substances. The results were expressed in mg/L.

### 3.5. Determination of Total Phenolics

Total phenolic content (TPC) was determined by the Folin–Ciocalteu colorimetric method [63] with some modifications. Strawberry wine was centrifuged at 4000× *g* (4 °C) for 15 min (5810 R, Eppendorf, Germany), and 25 μL of diluted supernates or standard solutions were mixed with 125 μL Folin–Ciocalteu reagent. After incubation for 5 min at 50 °C (HCM 100-Pro, Dargon, China), 100 μL Na_2_CO_3_ solution (75 g/L) was added and kept in the dark for 30 min (26 °C) before the absorbance was measured at 760 nm (Infinite 50, Tecan, Männedorf, Switzerland). The result was expressed as the gallic acid equivalent (GAE) in mg GAE/L, with the following standard curve regression equation: y = 0.0095x + 0.0257 (R^2^ = 0.999).

Total flavonoid content (TFC) was determined by the aluminum nitrate colorimetric method [64] with some modifications. Briefly, 1.0 mL of diluted strawberry wine was reacted with 0.3 mL of 50 g/L sodium nitrite for 6 min. Then, 0.3 mL of 100 g/L aluminum nitrate was added and maintained for 6 min. Finally, 2.4 mL of 40 g/L sodium hydroxide was added. The absorbance was measured at 510 nm after a reaction time of 15 min with a spectrophotometer (Specord 50, Analytic Jena, Jena, Germany). The result was expressed as rutin equivalent (RE) in mg RE/L, with the standard curve regression equation: y = 0.0024x + 0.0225 (R^2^ = 0.999).

Total anthocyanin content (TAC) was determined using the pH difference method [65] and expressed as cyanidin-3-O-glucoside chloride equivalent (CGE) in mg CGE/L. The 0.5 mL diluted strawberry wine and 2.0 mL of two different buffers (0.025 mol/L KCl pH 1.0 and 0.4 mol/L CH_3_COONa pH 4.5) were mixed. After incubation for 15.0 min in the dark, the absorbance was measured at 510 and 700 nm. The total anthocyanin was (mg CGE/L) = [(A/(ε × L)] × MW × DF, where A = (Abs510 − Abs700)pH1.0 − (Abs510 − Abs700)pH4.5, ε = 26,900 L/mol·cm (molar absorptivity of cyanidin-3-Oglucoside), where L is the correction factor for a 1 cm optic path length, MW is 449.2 g/mol (molecular mass of cyanidin-3-O-glucoside), DF is the dilution factor.

### 3.6. Determination of Antioxidant Capacity

The free radical scavenging capacity of DPPH was calculated by referring to the method of Liu et al. [64], with some modifications. First, 0.2 mL of the supernatant or standard solution was mixed with 4.8 mL DPPH solution (10 mM in methanol) and then left in the dark for 30 min. The inhibition against DPPH was measured at 517 nm (Infinite 50, Tecan, Switzerland). The standard curve was plotted with the free radical scavenging capacity of 10–100 μg/mL Vc to obtain the linear regression equation y = 0.0063x + 0.2059 (R^2^ = 0.999), and the ascorbic equivalent antioxidant capacity of grenadine was calculated as the AEAC (ascorbic equivalent antioxidant capacity, AEAC) in mg AEAC/L.

The cationic radical scavenging rate of ABTS was determined by referring to the method described by Re et al. [66], the standard curve was plotted with 10–100 μg/mL Vc scavenging ability of free radicals, the linear regression equation was obtained as y = 0.0138x + 0.0248 (R^2^ = 0.999), and the grenadine was diluted by appropriate multiples to calculate its AEAC in μg AEAC/L.

### 3.7. Determination of of Volatile Organic Compounds (VOCs)

VOCs were isolated using headspace solid-phase microextraction (HS-SPME) and analyzed by gas chromatography/mass spectrometry (GC/MS) according to the method modified by Zhang et al. [67]. A 5.0 mL sample was accurately pipetted into a 20 mL headspace vial, and 1.5 g NaCl was added to promote volatile component volatilization. 2-octanol was added as an internal standard (IS), and the final concentration in wine was 3.10 mg/L. The vial was sealed with a cap. After preconditioning at 45 °C for 10 min, the activated microextraction head (DVB/CAR/PDMS 50/30 μm, Supelco, Bellefonte, PA, USA) was stabbed into the headspace vial and enriched for 40 min with stirring (500 rpm). For desorption, the fiber was immediately injected into the GC/MS injector port and desorbed for 8 min (splitless mode).

Identification and quantification of aromas were performed using an Agilent 7890A-GC 5975C system (Agilent, Santa Clara, CA, USA). The column used was a 30 m × 0.25 mm × 0.25 μm DB-225 (Agilent, Santa Clara, CA, USA). The initial oven temperature was 40 °C, then increased at 2 °C/min to 160 °C and was kept at 160 °C for 2 min, and then increased at 2 °C/min to 230 °C and was kept at 230 °C for 2 min. The injector, transfer line, and ion trap temperatures were 250, 250, and 230 °C, respectively. Mass spectra were acquired in EI mode (70 eV) at 1 s/scan, full scan, with a range of 30–500 *m*/*z*. The carrier gas was helium (1 mL/min). The volatile compounds were identified by the retention time (RT) and by comparing their mass spectral data with those in the commercial mass spectral libraries of the NIST 1.1 mass spectral database, and a match degree of >700 was required for library comparison. The quantification was carried out by the internal standard method, and the calculation formula was as follows. The relative concentration of volatiles was calculated as the peak area of volatile/peak area of internal standard × concentration of internal standard. The results were expressed in μg/L.

### 3.8. Odor Activity Values (OAVs)

OAVs were calculated by dividing the calculated relative concentration of the volatile compound by its odor threshold.

### 3.9. Statistical Analysis

All the experiments were conducted three independent times. One-way analysis of variance (ANOVA) was carried out with Duncan’s multiple range test to determine the significant differences (*p* < 0.05) using SPSS statistical software 21.0 (IBM Corporation, New York, NY, USA). The heatmap was plotted using HemI 1.0.3.7. Principal component analysis (PCA) was performed using Origin 2021.

## 4. Conclusions

This research investigated the influence of pre-treatment methods on the physicochemical properties and volatile profiles of strawberry wines. Strawberry wines fermented from the juice after maceration had more desirable qualities, such as less methol content compared with pulp-fermented wines, and more total phenols, enhanced antioxidant activity, richer essential terpenoids, and fatty acid ethyl esters with pleasant floral and fruity notes compared with juice-fermented wines without maceration, in addition to the lighter color. In particular, thermovinification at 50 °C and enzymatic maceration provided more ethyl caproate and ethyl caprylate, which contributed apple, pineapple, and strawberry flavors to the strawberry wine. However, the two wines differed in that the 50J wine displayed a slightly yellow hue, as well as a rose and cheese aroma, with similar levels of phenylethyls, acids, and γ-decanolactone, while the PJ wine displayed more of a red hue and southern fruit traits like orange and lemon. Unfavorably, the methanol content substantially rose as a result of the addition of commercial pectinase. All things considered, juice thermovinification at 50 °C was shown to be a safe fermentation technique with an acceptable content of methanol that enhanced the nutritive and aromatic characteristics of strawberry wines.

## Figures and Tables

**Table 1:** Methods of pre-fermentation treatment.

Number	Name and Abbreviation of Fermentation Method	Fermentation Raw Material	Maceration Time/h	Maceration Temperature/°C	Pectinase
A	control vinification of juice pressed from must without pretreatment (CJ)	juice	/	/	/
B	vinification of juice pressed from must macerated at normal temperature (NJ)	juice	3	25	/
C	vinification of juice pressed from must macerated at 50 °C (50J)	juice	3	50	/
D	vinification of juice pressed from must macerated at 50 °C with pectinase (PJ)	juice	3	50	added
E	vinification of must macerated at normal temperature (NM)	must	3	25	/
F	vinification of must macerated at 50 °C with pectinase (PM)	must	3	50	added

Notes: “/” indicates that the process was not performed.

**Table 2:** Chemical indicators and color attributes of strawberry wines.

	CJ	NJ	50J	PJ	NM	PM
Alcoholic content (% *v*/*v*)	12.17 ± 0.23 b	12.3 ± 0.00 b	12.3 ± 0.00 b	12.5 ± 0.17 a	12.27 ± 0.1 b	12.3 ± 0.00 b
Sugar-free extract (g/L)	16.48 ± 0.20 c	18.72 ± 0.3 b	18.51 ± 0.26 b	17.48 ± 1.37 b	19.39 ± 0.23 a	19.32 ± 0.39 a
Total sugar (g/L)	1.83 ± 0.11 d	2.51 ± 0.19 bc	2.73 ± 0.21 b	5.32 ± 0.42 a	2.26 ± 0.19 c	2.23 ± 0.15 c
Methanol (mg/L)	8.87 ± 1.01 e	67.12 ± 4.58 c	72.43 ± 2.14 c	136.72 ± 11.5 a	88.16 ± 7.52 b	134.08 ± 2.65 a
Titratable acidity (g/L)	7.72 ± 0.05 e	8.11 ± 0.05 bc	8.04 ± 0.09 c	7.87 ± 0.05 d	8.54 ± 0.07 a	7.90 ± 0.08 d
Malic acid(g/L)	1.21 ± 0.05 a	1.11 ± 0.04 b	0.98 ± 0.01 de	0.92 ± 0.07 e	1.07 ± 0.05 bc	1.01 ± 0.02 cd
Lactic acid(g/L)	0.25 ± 0.05 b	0.17 ± 0.04 b	0.22 ± 0.01 b	0.24 ± 0.00 b	0.38 ± 0.07 a	0.18 ± 0.02 b
Acetic acid(g/L)	0.12 ± 0.02 c	0.10 ± 0.03 c	0.11 ± 0.01 c	0.57 ± 0.09 b	0.97 ± 0.34 a	0.35 ± 0.03 bc
Citric acid(g/L)	4.47 ± 0.02 bc	4.53 ± 0.03 bc	4.71 ± 0.04 ab	4.34 ± 0.12 c	4.93 ± 0.43 a	4.88 ± 0.02 a
Succinic acid(g/L)	0.20 ± 0.01 f	0.49 ± 0.01 cd	0.52 ± 0.03 b	0.42 ± 0.03 e	0.61 ± 0.02 a	0.50 ± 0.00 bc
L*	16.86 ± 0.06 e	17.25 ± 0.01 d	17.58 ± 0.04 c	16.53 ± 0.03 f	18.09 ± 0.04 b	18.44 ± 0.02 a
a*	28.05 ± 0.14 a	18.00 ± 0.05 c	15.60 ± 0.02 e	21.90 ± 0.03 b	16.35 ± 0.02 cd	15.60 ± 0.07 e
b*	26.70 ± 0.15 b	25.05 ± 0.02 bc	26.85 ± 0.05 b	29.70 ± 0.03 a	28.95 ± 0.04 a	29.10 ± 0.09 a
c*	38.72 ± 0.21 a	30.85 ± 0.02 d	31.05 ± 0.03 d	36.90 ± 0.02 b	33.25 ± 0.02 c	33.02 ± 0.11 c
h	43.56 ± 0.71 c	54.24 ± 1.59 b	59.85 ± 1.11 a	53.60 ± 0.81 b	60.54 ± 0.99 a	61.80 ± 0.77 a

Note: Different lowercase letters in the same row represent significant differences (one-way ANOVA–Dunace, *p* < 0.05).

**Table 3:** OVAs of strawberry wines.

No.	Compounds	RT/min	CJ	NJ	50J	PJ	NM	PM	Descriptor	Thresholds (µg/L)
A1	1-Hexanol	10.85	0.05	0.09	0.06	0.06	0.12	0.09	Resin, green	8000 [31,32]
B1	γ-Decanolide	46.30	42.72	30.12	45.16	32.27	60.19	24.35	Peach	11 [33]
D1	Linalool	21.18	37.02	39.84	44.33	38.25	52.78	34.45	Floral, fruity, musky	15 [32,34]
D2	Geranyl acetone	24.18	0.09	0.32	0.13	0.16	0.81	0.05	Tropical fruits	60 [32]
D3	α-Terpinol	27.00	0.43	0.46	0.43	0.43	0.52	0.26	Pleasant, sweet, anise	250 [31,35]
D4	Myrtenol	28.76	0.00	2.95	2.27	2.63	1.13	0.28	Floral, mint	7 [36]
D6	(E,E)-Farnesol	34.66	0.39	0.33	0.35	0.61	0.33	0.35	Lemon, anise, floral, honey, pollen, raspberry	100 [35]
D8	Trans-nerolidol	38.99	0.15	0.09	0.14	0.40	0.30	0.29	Floral, fruity, orange, light flavor	700 [37]
E1	Methyl salicylate	28.33	0.16	0.29	0.32	0.80	0.40	0.52	Holly	40 [38]
F1	1-Butanol, 3-methyl-	2.34	0.39	0.42	0.38	0.27	0.46	0.46	Whiskey, harsh, bitter	30,000 [34,35]
F3	1-Octanol	20.85	0.47	0.56	0.68	0.42	1.97	1.70	Roses, citrus	120 [37]
F4	2-Nonanol	20.68	0.00	0.07	0.07	0.10	0.10	0.11	Fat wax, rose, citrus	50 [37]
G1	1-Butanol, 3-methyl-, acetate	5.05	2.45	2.84	3.27	2.50	0.75	0.47	Banana	1500 [39]
G3	Acetic acid, hexyl ester	13.43	51.50	71.67	55.06	65.14	6.21	3.79	Apple, banana, pear	10 [37]
H1	Hexanoic acid, ethyl ester	12.09	112.47	210.67	241.02	246.77	152.57	125.71	Apple, strawberry	14 [31,32]
H4	Heptanoic acid, ethyl ester	17.11	0.04	0.11	0.12	0.11	0.09	0.06	Pineapple, fruity	220 [37]
H6	Octanoic acid, ethyl ester	21.66	759.46	1007.75	1180.29	1333.33	440.28	234.82	Pineapple, apple, brandy	2 [37]
H11	Decanoic acid, ethyl ester	29.77	1.78	3.16	4.18	5.70	1.76	0.72	Pear, brandy	200 [32,37]
H12	Ethyl 9-decenoate	30.98	0.75	4.73	7.59	6.72	2.56	0.22	Fruity	100 [40]
H14	Dodecanoic acid, ethyl ester	36.97	0.04	0.06	0.33	0.25	0.07	0.02	Oil, floral	400 [40]
L1	Styrene	8.08	5.26	6.85	7.12	6.34	5.09	4.36	Flowery	65 [41]
L2	Benzaldehyde	19.19	0.42	0.10	0.23	0.13	0.09	0.09	Almond	2000 [41]
L3	Acetophenone	25.17	1.21	0.33	1.05	0.57	1.03	0.69	Almond	65 [42]
L5	Phenylethyl Alcohol	30.40	0.20	0.23	0.28	0.10	0.35	0.08	Honey, rose	14,000 [31,32]
L6	Acetic acid, 2-phenylethyl ester	31.95	7.51	4.61	6.96	2.82	2.45	0.94	Floral	250 [37]
L7	Benzoic acid, ethyl ester	26.44	0.05	0.11	0.14	0.08	0.32	0.10	Holly oil and fruit	575 [32]
L8	Benzeneacetic acid, ethyl ester	31.25	0.03	0.10	0.10	0.06	0.10	0.05	Floral, honey	73 [37]
L9	Benzenepropanoic acid, ethyl ester	34.97	0.45	0.24	0.86	1.00	1.53	0.79	Floral	18.5 [43]
L10	2-Propenoic acid, 3-phenyl-, ethyl ester	41.09	228.80	424.48	516.99	508.17	423.87	155.73	Honey, cinnamon	1.1 [34]
I1	Octanoic acid, methyl ester	18.83	0.01	0.06	0.06	0.14	0.01	0.03	Orange	200 [44]
J2	Hexanoic acid	23.60	1.16	0.56	0.91	0.57	1.36	0.44	Cheese	420 [39]
J3	Octanoic acid	31.63	1.65	0.37	0.77	0.38	0.41	0.09	Fatty acid	500 [39]
K3	Decanal	24.87	1.07	1.32	0.81	0.30	0.42	0.67	Orange peel	10 [39]
	Total		1177.33	1741.68	2029.59	2182.76	1044.24	532.65		

## Data Availability

The original contributions presented in the study are included in the article/Supplementary Material, further inquiries can be directed to the corresponding authors.

## Supplementary Materials

The following supporting information can be downloaded at: https://www.mdpi.com/article/10.3390/molecules29092045/s1, Table S1: Relative content of volatile compounds in strawberry wines.
